# Local Production of Activated Factor X in Atherosclerotic Plaque Induced Vascular Smooth Muscle Cell Senescence

**DOI:** 10.1038/s41598-017-17508-6

**Published:** 2017-12-07

**Authors:** Fumihiro Sanada, Jun Muratsu, Rei Otsu, Hideo Shimizu, Nobutaka Koibuchi, Kazutaka Uchida, Yoshiaki Taniyama, Shinichi Yoshimura, Hiromi Rakugi, Ryuichi Morishita

**Affiliations:** 1 Department of Clinical Gene Therapy, Osaka University Graduate School of Medicine, Suita, Osaka, 565-0871 Japan; 2 Department of Geriatric and General Medicine, Osaka University Graduate School of Medicine, Suita, Osaka, 565-0871 Japan; 3 Departments of Pharmacology and Molecular Therapeutics, Kumamoto University Graduate School of Medical Sciences, Kumamoto, Japan; 4 Department of Neurosurgery, Hyogo College of Medicine, Hyogo, Japan

## Abstract

Our previous study demonstrated that coagulation factor Xa (FXa) induced endothelial cell senescence, resulting in inflammation and impaired angiogenesis. This mechanism is dictated through protease-activated receptors, PARs, insulin-like growth factor-binding protein 5 (IGFBP-5), and p53. Activation of PARs contributes to the pathophysiology of several chronic inflammatory diseases, including atherosclerosis. Thus, we speculated that similar mechanism might participate in the progression of atherosclerotic plaques. In the present study, we successfully identified the cells that produced FX/Xa in atherosclerosis using human atherosclerotic plaques obtained from carotid endarterectomy. *In situ* hybridization for FX revealed that FX was generated in vascular smooth muscle cells (VSMC), inflammatory cells, and endothelial cells. Then, we examined the effects of FXa on the growth of VSMC *in vitro*. The present study revealed that chronic FXa stimulation significantly induced the senescence of VSMC with concomitant upregulation of IGFBP-5 and p53. Inhibition of FXa signaling with rivaroxaban or knock down of IGFBP-5 significantly reduced FXa-induced VSMC senescence and inflammatory cytokine production. Finally, we confirmed that FXa and IGFBP-5 are co-distributed in atherosclerotic plaques. In conclusion, induction of senescence of VSMC induced by locally produced FX/Xa may contribute to the progression of atherosclerosis.

## Introduction

Great advances have been made in anti-coagulation therapy for cardiovascular disease in recent years^1,2^. Direct targeting of activated coagulation factor X (FXa) is a relatively new strategy for the treatment of atrial fibrillation and deep vein thrombosis^3,4^. Currently, three drugs from the class of direct Xa inhibitors are marketed worldwide. Rivaroxaban was the first approved FXa inhibitor to become commercially available in Europe and Canada in 2008. Intriguingly, recent findings suggest that FXa and thrombin have non-hematologic functions beyond the coagulation cascade^5–7^. This mechanism operates in several chronic inflammatory diseases through protease-activated receptors, PARs^7,8^. FXa significantly increases inflammatory cytokine production in fibroblasts, macrophages and endothelial cells (ECs)^9^. In support of this notion, a clinical trial demonstrated that rivaroxaban reduced the risk of the composite end point of death from cardiovascular causes, myocardial infarction, or stroke in patients with a recent acute coronary syndrome^10^. Based on these results, FXa inhibition might also have a beneficial role in patients with atherosclerosis.

In this study, we demonstrated that FX/Xa was produced in vascular smooth muscle cells (VSMCs), inflammatory cells, and endothelial cells (ECs) in human atherosclerotic plaques by *in situ* hybridization. We also identified colocalization of FXa in VSMCs and inflammatory cells by immunohistochemical analysis. In addition, the present study revealed that continuous FXa stimulation *in vitro* upregulated senescence markers, such as p53, and increased the fraction of senescence-associated β-galactosidase (SA-β gal)-positive cells through the activation of PAR1/2 and insulin-like growth factor binding protein 5 (IGFBP-5). These senescent VSMCs increased inflammatory cytokine production, acquiring the so-called senescent associated secretary phenotype (SASP)^11^. However, rivaroxaban or genetic knockdown of PAR1, PAR2, and IGFBP-5 attenuated FXa-induced VSMC senescence and inflammatory cytokine expression. Our data suggest that locally produced FXa induced SMC senescence, leading to chronic inflammation in atherosclerosis.

## Results

### FX production in atherosclerotic plaques

**Figure 1:**
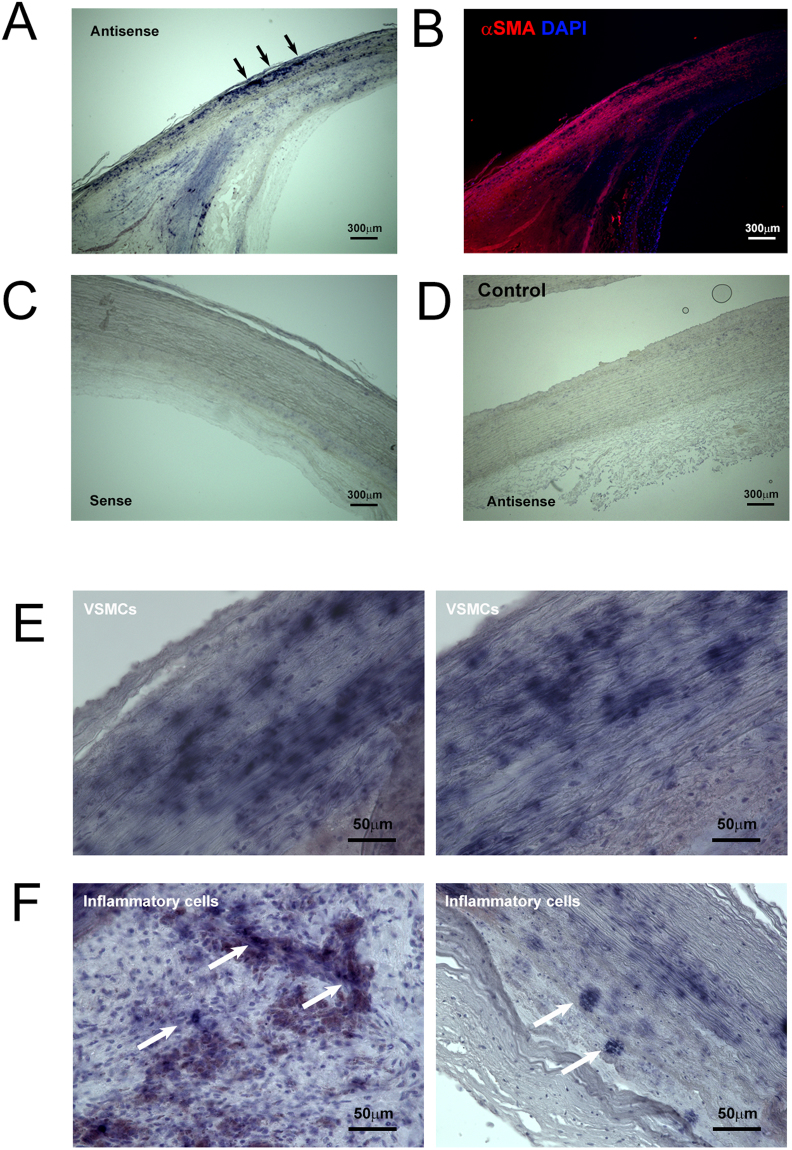
*In situ* hybridization of coagulation FX in human atherosclerotic plaques. (**A**) *In situ* hybridization of human atherosclerotic plaques obtained from the patients subjected to carotid endarterectomy with an antisense probe to human FX. (**B**) This panel shows αSMA and DAPI staining. (**C**) The sense probe strand shows no specific hybridization. (**D**) Control carotid artery samples with antisense probe demonstrated much weaker signals compared to the samples from patients. (**E**,**F**) A higher-magnification image shows strong positive staining in VSMCs (**E**) and inflammatory cells (**F**).

First, we examined local production of FX/Xa in atherosclerotic plaques in human carotid endarterectomy samples. FX mRNA expression was assessed by *in situ* hybridization in relation to the simultaneous antigen staining of alpha-smooth muscle actin (αSMA). FX mRNA was mainly detected in cells positive for αSMA (Fig. 1A and B, arrow), visible as dots in the neointima (Supplement Fig. 1, arrow), with some staining in ECs (Supplement Fig. 1, arrowhead). Much weaker signals were observed in samples with sense probe (Fig. 1C) and control samples with antisense probe (Fig. 1D). Higher magnification clearly showed strong FX mRNA signals in VSMCs (Fig. 1E) and in the accumulated inflammatory cells (Fig. 1F). Additionally, as shown in Supplement Fig. 2, immunohistological analysis showed positive staining for FX/FXa in the VSMC layer (white arrow) and inflammatory cells (black arrow). These data provided the evidence that FX was locally synthesized and activated in atherosclerotic plaques, although FX from the liver might also be involved and distributed within the plaque. Given FX mRNA is present and FX is activated locally in atherosclerotic plaque, intrinsic and extrinsic coagulation factors that activate FX was investigated by *in situ* hybridization. As show in Supplement Fig. 3, strong FIII, FVIII, and FX mRNA signals was detected in inflammatory cells (arrow head) and VSMCs (black arrow), Although FVII signal was relatively weak, it was detected in inflammatory cells and VSMCs. In support of this notion, we observed FIII, FVII, FVIII, and FX mRNA expression *in vitro* in human aortic SMCs, THP-1 monocytes, and human aortic endothelial cells (Supplement Fig. 4). FX mRNA expression in human aortic VSMCs was increased by the stimulation of angiotensin (Ang) II (200 nM) and FXa (50 nM) stimulation (Supplementary Figure 5). These data imply that coagulation FX is produced and activated locally in human atherosclerosis.

### Chronic FXa treatment induced senescence of human aortic VSMCs through IGFBP-5

**Figure 2:**
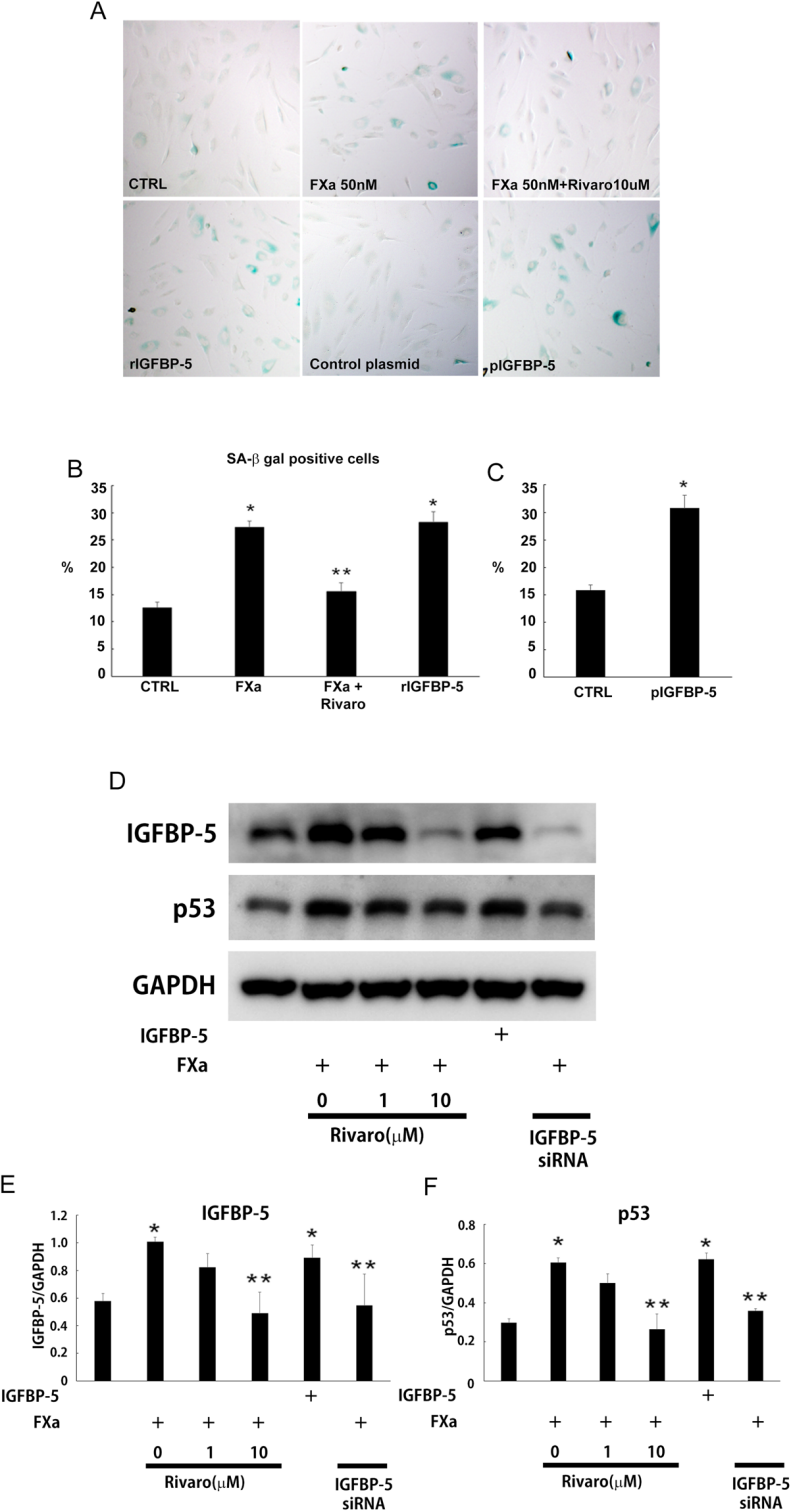
Induction of SMC senescence by FXa through IGFBP-5. (**A**–**C**) Human aortic VSMCs were treated every other day for 14 days with FXa (50 nM), FXa (50 nM) with rivaroxaban (10 μM), or recombinant IGFBP-5 (rIGFBP-5, 100 ng/mL). Additionally, IGFBP-5 was overexpressed in human aortic VSMCs using pIGFBP5. (**A**) Senescent cells were detected by senescence associated β-galactosidase staining (SA-β-gal staining). (**B**) The fractions of SA-β gal-positive VSMCs. *, **P < 0.05 vs. control (CTRL) and FXa, respectively. n = 10. (**C**) Over-expression of IGFBP-5 increased the fractions of SA-β Gal-positive VSMCs. *P < 0.05 vs. control (CTRL). n = 10. (**D**–**F**) FXa significantly increased the expression of IGFBP-5 and p53, and similarly, rIGFBP-5 up-regulated p53 expression in human aortic VSMCs. However, rivaroxaban or IGFBP-5 siRNA remarkably reduced FXa-induced IGFBP-5 and p53 up-regulation. *, **P < 0.05 vs. control and FXa, respectively. n = 4.

Next, we assessed the functional role of FXa beyond coagulation in human aortic VSMCs. We previously demonstrated that FXa induced EC senescence through the IGFBP-5-p53 pathway, resulting in impaired tissue repair^12^. Thus, human aortic VSMCs were treated with FXa (50 nM) every other day for 14 days, and analyzed for cellular senescence markers. At day 14, the fraction of SA-β gal positive VSMCs was significantly increased in response to FXa treatment (Fig. 2A–C). Similarly, recombinant IGFBP-5 (rIGFBP-5) treatment or IGFBP-5 overexpression remarkably increased the fraction of senescent cells. However, rivaroxaban or direct FXa inhibition attenuated FXa-induced SA-β gal activity. Additionally, VSMCs treated with FXa exhibited significantly increased the expression of IGFBP-5 and p53, and rIGFBP-5 treatment resulted in p53 upregulation (Fig. 2D–F). Rivaroxaban treatment significantly reduced FXa-induced IGFBP-5 and p53 upregulation in VSMCs. Moreover, IGFBP-5 knockdown by siRNA substantially reduced FXa**-**induced p53 upregulation. These results, together with our previous study^12^, suggest that FXa might induce cellular senescence through the IGFBP-5-p53-dependent pathway in human aortic VSMCs.

### FXa-induced senescent VSMCs increased inflammatory cytokine expression and decreased the proliferation

**Figure 3:**
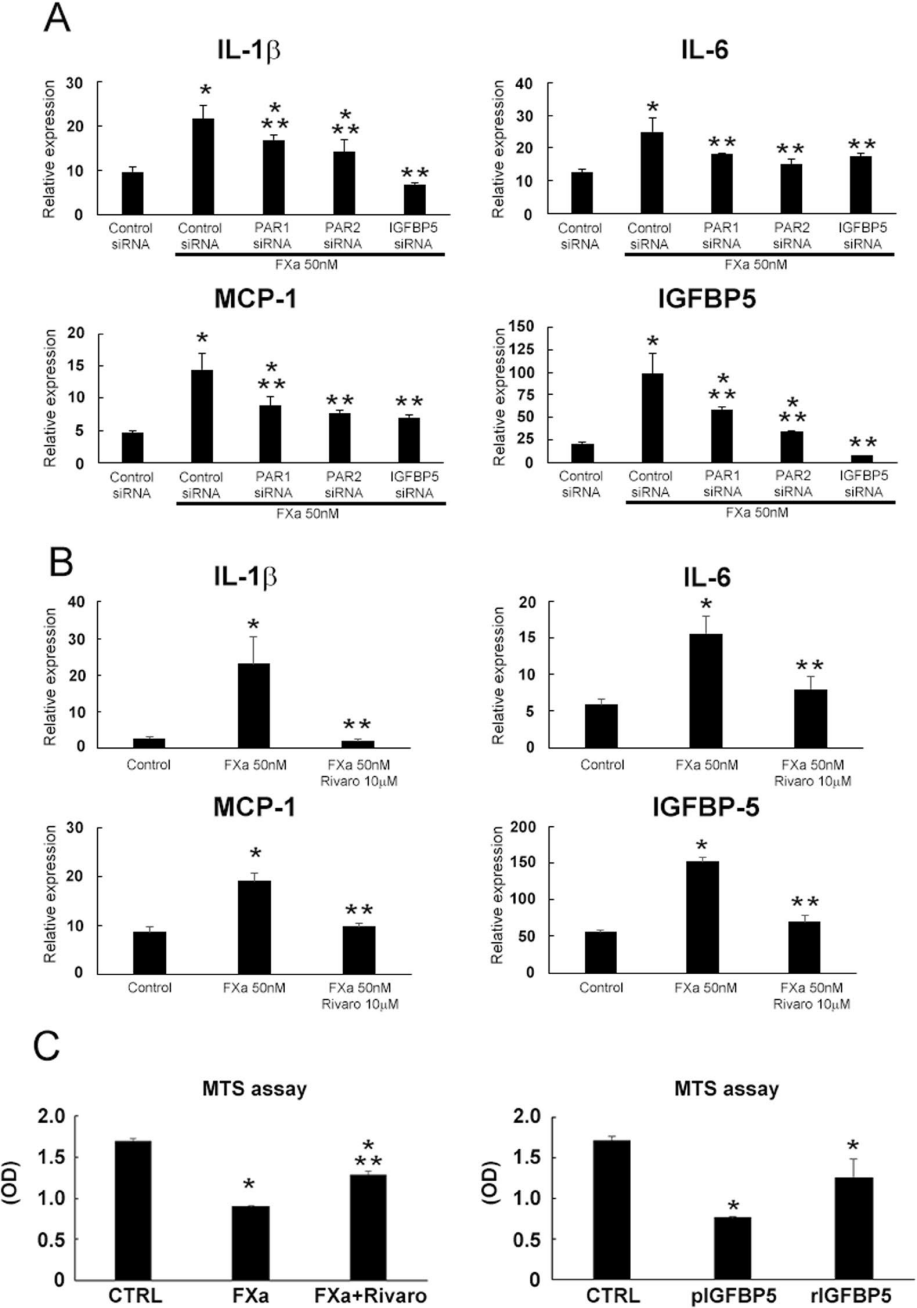
Senescence-associated inflammatory responses and impaired the proliferation. (**A**) Relative mRNA expression of IL-1β/GAPDH, IL-6/GAPDH, MCP-1/GAPDH, and IGFBP-5/GAPDH in FXa treated human aortic VSMCs. *, **P < 0.05 vs. control siRNA and control siRNA + FXa 50 nM, respectively. n = 3. (**B**) FXa-induced inflammatory cytokine expression was reduced by the direct FXa inhibitor, ribaroxban. *, **P < 0.05 vs. control and FXa 50 nM, respectively. n = 5. (**C**) The ability of FXa-treated human aortic VSMCs to proliferate was measured by MTS assay. *, **P < 0.05 vs. control (CTRL) and FXa (50 nM), respectively. n = 6.

Accumulating data have provided the evidence that senescent cells could cause the harmful effects on the tissue microenvironment. Among these effects, acquisition of the senescence-associated secretory phenotype (SASP) sustains low-grade inflammation, promoting several chronic inflammatory diseases^11^. Thus, we compared the expression of inflammatory cytokines and the ability to proliferate with or without chronic FXa treatment. As shown in Fig. 3A and B, senescent human aortic VSMCs treated with FXa exhibited a significant increase in the expression of inflammatory mediators, such as IL-1β, IL-6, and MCP-1, along with IGFBP-5, while PAR1, PAR2, IGFBP-5 siRNA, or rivaroxaban significantly decreased the expression of these cytokines. Similarly, VSMCs treated for 14 days with FXa or rIGFBP-5 as well as VSMCs overexpressing IGFBP-5 demonstrated the impaired proliferation *in vitro* (Fig. 3C). However, addition of rivaroxaban attenuated the inhibition of the proliferation of FXa-treated VSMCs. These data strongly suggest that continuous FXa may induce SASP phenotype and impair the proliferation of human aortic VSMCs, similar to ECs treated with FXa.

### Co-localization of FXa and IGFBP-5 in atherosclerotic plaques

**Figure 4:**
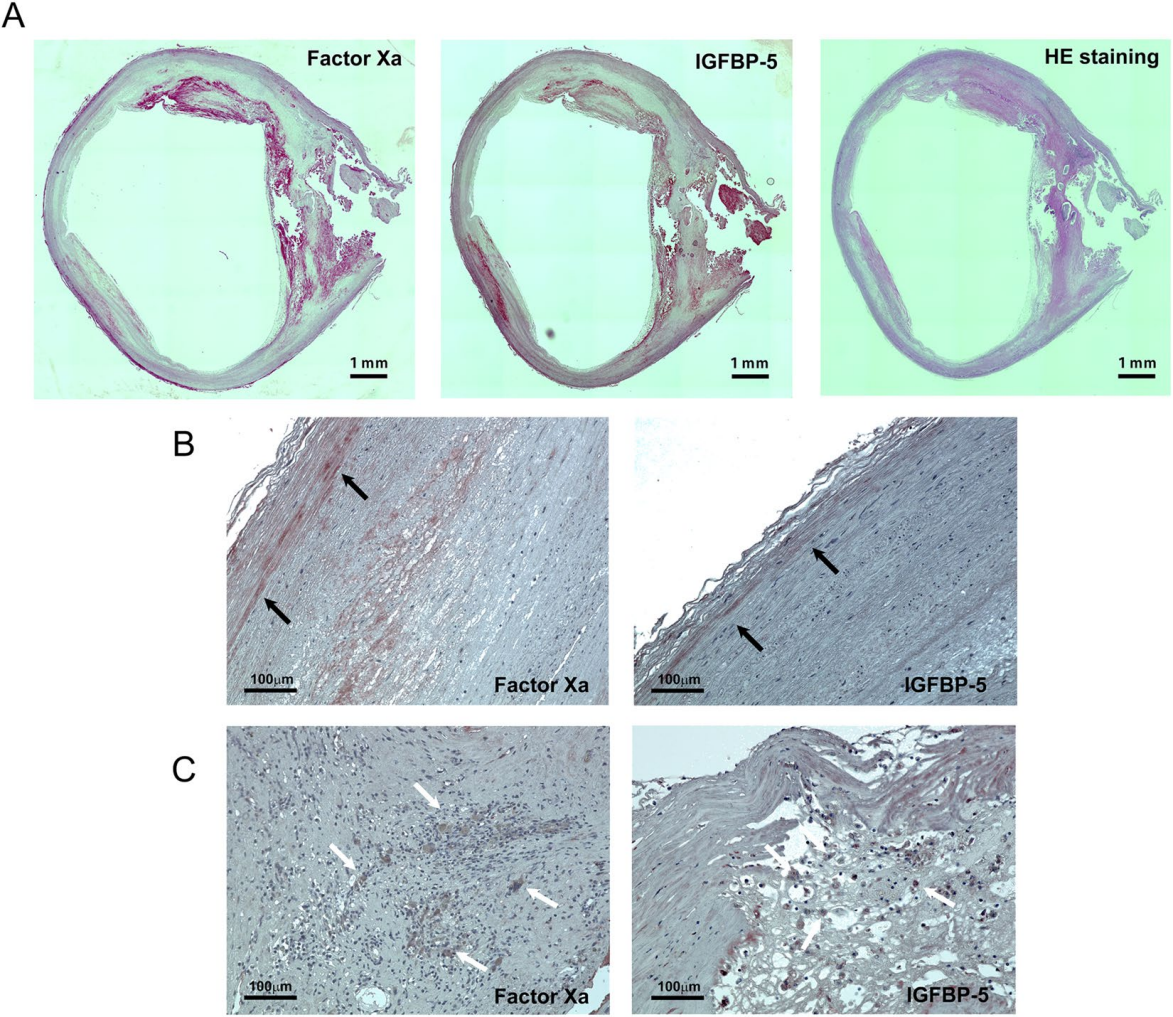
Distribution of FXa and IGFBP-5 staining area in human atherosclerotic plaques. (**A**) Human atherosclerotic plaques were stained with FXa (left), IGFBP-5 (middle), and Hematoxylin-Eosin staining (right). (**B**,**C**) Higher magnification of the FXa and IGFBP-5 positive area in VSMCs (**B**, black arrow) and inflammatory cells (**C**, white arrow).

To support the clinical relevance of our *in vitro* findings, serial sections from human atherosclerotic plaques were stained with FX/FXa antibody, IGFBP-5 antibody, or Hematoxylin-Eosin (Fig. 4A). The FX/FXa and IGFBP-5-positive areas were similarly distributed, and the expression of both FX/FXa and IGFBP-5 was observed in VSMCs (Fig. 4B, black arrow) and inflammatory cells (Fig. 4C, white arrow) within the plaque. These staining patterns suggest that locally produced FXa in VSMCs and inflammatory cells might induce IGFBP-5 expression, resulting in the chronic inflammation and cellular senescence in human atherosclerosis.

## Discussion

Normally, anti-coagulant systems dominate coagulation systems, preventing thrombotic complications in physiological condition^13^. However, this balance collapses in the setting of chronic inflammatory diseases such as atherosclerosis. Uncontrolled coagulation activity overwhelms the anti-coagulation system, introducing tissue inflammation, fibrosis, and cellular senescence, leading to tissue remodeling through protease-activated receptors, PARs^5,14,15^. Among coagulation factors, FXa signaling is of current interest, not only as the first member of the final common pathway or thrombin pathway, but also for its non-hematologic functions beyond blood coagulation. FXa proteolytic activities were found to be significantly increased in early atherosclerotic lesions compared with lesions at a later stage^16^. In contrast, rivaroxaban, a direct FXa inhibitor, has been reported to reduce the risk of recurrent atherothrombotic events in patients with acute coronary syndrome^10,17^. These data imply an important role for FXa-mediated cellular effects in the initial development of atherosclerosis that goes beyond thrombus formation. However, the origin and biological effects of FXa in atherosclerotic plaques is still unclear.

According to the standard paradigm, synthesis of coagulation FX is primarily restricted to the liver^18,19^. However, the expression of coagulation FX is much more complex than previously thought, as our *in situ* hybridization data showed that FX was also locally produced in VSMCs, ECs, and inflammatory cells (Fig. 1A–F). Our *in vitro* experiments confirmed not only FX mRNA, but also FIII, FVII, and FVIII expression in human aortic VSMCs (Supplementary Figure 3). Consistently, *in vitro* experiments documented that coagulation factor III, VII, VIII, IX, and X mRNA were detectable in human aortic VSMCs, THP-1 monocytes, and human aortic ECs (Supplementary Figure 4). These observations indicates that several intrinsic and extrinsic coagulation factors are locally synthesized and activates coagulation factor X. Moreover, our previous reports documented that FXa stimulation on ECs enhanced cellular senescence through the IGFBP-5-p53 pathway^12^. Thus, we speculate that this intricate regulation of FX expression in atherosclerosis would induce SMC senescence and aggravate inflammation. As expected, FXa stimulated both PAR1 and PAR2, leading to IGFBP-5-p53 pathway activation in human aortic VSMCs (Figs 2D–F and 3A). Consequently, FXa increased the fraction of SA-β gal-positive VSMCs and promoted inflammatory cytokine expression (Figs 2A,B, and 3B). By blocking FXa-mediated PAR-IGFBP-5-p53 signaling with rivaroxaban, PAR siRNA or IGFBP-5 siRNA, IGFBP-5 expression, subsequent cellular senescence, and production of inflammatory mediators were remarkably reduced. These data confirmed that FXa stimulation on VSMCs enhanced cellular senescence and subsequent inflammatory cytokine production through the IGFBP-5-p53 pathway. Among IGFBPs, IGFBP-5 has been shown to inhibit the growth of breast cancer cells^20^, ECs^21,22^, and fibroblasts^23,24^ by inducing cellular senescence. Moreover, atherosclerotic arteries in humans exhibited strong IGFBP-5-positive staining along intimal plaques^21^. Consistently, our immunostaining also revealed the potent IGFBP-5 positive staining in atherosclerotic plaque, which was well co-localized with FX/FXa expression (Fig. 4A–C). These data indicated that FXa functions as one of the upstream effectors of IGFBP-5 in VSMCs, inducing cellular senescence and inflammation in atherosclerosis. Of note, it is well known that the plasma concentrations of many coagulation factors increase in healthy humans in parallel with the physiological processes of aging^25,26^. Our data imply that this heightened pro-coagulant status with age may reflect ongoing inflammatory and cell senescence processes which is known as normal phenomenon of progressive life.

In summary, the present study clearly demonstrated that FX/FXa was locally synthesized in VSMCs, ECs, and inflammatory cells in human atherosclerosis. Activated FX initiated the senescence in VSMCs and ECs, and subsequent inflammatory cytokine production, the SASP phenotype, which might be occurring in atherosclerotic plaques. Rivaroxaban, an FXa inhibitor, significantly inhibited FXa-induced activation of IGFBP-5 and p53, and prevented cellular senescence and inflammation. This results might support the ATLAS ACS 2–TIMI 51 clinical trial that in patients with a recent acute coronary syndrome, rivaroxaban reduced the risk of the composite end point of death from cardiovascular causes, myocardial infarction, or stroke.

## Materials and Methods

### Ethical Statement

All methods and experiments were performed in accordance with the approved guidelines of Osaka University Medical School. The atherosclerotic vascular samples (n = 2) used in this study were obtained from the patients subjected to carotid endarterectomy. Vascular surgical procedures were performed at the Department of Neurosurgery at The Hospital of Hyogo College of Medicine. This study was approved by the Ethics Committees of both Osaka University (approved number; 13–415) and the Hospital of Hygo College of Medicine. Informed consent was obtained from the patients. Control carotid artery samples (n = 2) were obtained from Bizcom Japan (Tokyo, Japan).

### Reagents and antibodies

Human FXa was purchased from BioVision Inc, California, USA. Rivaroxaban was donated from Bayer pharma AG, Leverkusen, Germany. IGFBP-5 antibody was from R&D Systems, Minnesota, USA. p53 antibody was obtained from Cell Signaling, technology, Massachusetts, USA. IGFBP-5 plasmid (pcDNA3-IGFBP5-V5) was purchased from Addgene, Cambridge, MA, USA, and siRNA for PAR1/2 and IGFBP-5 were from Santa Cruz Biotechnology, Inc. Texas, USA. Human recombinant IGFBP-5 was from R&D Systems.

### *In situ* hybridization

*In situ* hybridization for human FX message was performed on 10 μm paraffin serial sections using DIG-labeled riboprobe transcribed from human FIII, FVII, FVIII, and FX cDNA. Hybridization with sense probe was performed in parallel as a negative control.

### Cell culture

Human aortic VSMCs (passage 5 to 9) purchased from Lonza were cultured in SmGM-2, Smooth Muscle Growth Medium-2 (Clonetics, Walkersville, Maryland, USA). Following serum starvation (0.5% FBS), human aortic VSMCs were stimulated with FXa (50 nM) with or without rivaroxaban (10 μM) every other day for 14 days. Over-expression of IGFBP-5 or siRNA knockdown experiments were performed for 10 days in human aortic VSMCs. Human aortic endothelial cells were purchased from Lonza and THP-1 monocyte was from ATCC.

### Proliferation assay

Mitogenic activity was measured with the MTS assay kit, according to the manufacturer’s instruction (Promega, Madison, Wisconsin, USA).

### Isolation of total RNA and RT-PCR

Total RNA was isolated using the RNeasy Mini Kit (QIAGEN, Hilden, Germany). DNase-treated total RNA was reverse-transcribed with the High-Capacity cDNA Reverse Transcriptase Kit (Applied Biosystems, Foster City, CA, USA) to produce complementary DNA (cDNA). Reverse transcription-generated cDNA encoding the target genes was amplified and quantified by the ViiA-7™ real-time PCR system (Applied Biosystems, Foster City, CA, USA) using the primer sets shown below.

### Human IL-1β;

Forward 5′-TACAGTGGCAATGAGGATGAC-3′

Reverse 5′-GTCGGAGATTCGTAGCTGGAT-3′

### Human IL-6;

Forward 5′-TGACAAACAAATTCGGTACATCCT-3′

Reverse 5′-AGTGCCTCTTTGCTGCTTTCAC-3′

### Human MCP-1;

Forward 5′-AGTCTCTGCCGCCCTTCTGTG-3′

Reverse 5′-TGCTGCTGGTGATTCTTCTAT-3′

### Human IGFBP-5;

Forward 5′-ACCCAGTCCAAGTTTGTCGG-3′

Reverse 5′-CGTCAACGTACTCCATGCCT-3′

### Human Factor III

Forward 5′-CAGAGTGTGACCTCACCGAC-3′

Reverse 5′-GTCCGAGGTTTGTCTCCAGG-3′

### Human Factor VII

Forward 5′-AGTACTGCAGTGACCACACG-3′

Reverse 5′-CAATTCGGCCTTGGGGTTTG-3′

### Human Factor VIII

Forward 5′-CTCCCTGGCTTGCCTTCTAC-3′

Reverse 5′-AATTGGATGCACCCTCCTGG-3′

### Human Factor IX

Forward 5′-TGACCGAGCCACATGTCTTC-3′

Reverse 5′-GGGTCCCCCACTATCTCCTT-3′

### Human Factor X;

Forward 5′-GGAGGTGGTCATCAAGCACA-3′

Reverse 5′-TCACAATCCCCGTCTTCTGC-3′

### Western Blot Analysis

Western blotting was performed as previously described^12^.

### Statistical Analysis

All statistical analyses were performed using the JMP statistics software package. Values are expressed as the means ± SE. ANOVA and t-test followed by the Tukey-Kramer adjustment for multiple comparisons were used to evaluate differences among more than two groups.

## Electronic supplementary material

Supplementary information
