# Supplementary material for: Local Production of Activated Factor X in Atherosclerotic Plaque Induced Vascular Smooth Muscle Cell Senescence
Source: Sci Rep. 2017 Dec 7;7:17172. doi: 10.1038/s41598-017-17508-6 (PMC5719421; doi:10.1038/s41598-017-17508-6)
Supplement: Supplementary file 1 — Supplementary information [file 41598_2017_17508_MOESM1_ESM.pdf]

Supplementary information for

Local Production of Activated Factor X in Atherosclerotic Plaque Induced Vascular Smooth Muscle Cell Senescence

Fumihiro Sanada[1], Jun Muratsu[1, 2], Rei Otsu[1], Hideo Shimizu[1], Nobutaka Koibuchi[3], Kazutaka Uchida[4], Yoshiaki Taniyama[1, 2], Shinichi Yoshimura[4], Hiromi Rakugi[2], Ryuichi Morishita[1]

[1]Department of Clinical Gene Therapy, [2]Department of Geriatric and General Medicine, Osaka University Graduate School of Medicine, Suita, Osaka 565-0871, Japan
[3]Departments of Pharmacology and Molecular Therapeutics, Kumamoto University Graduate School of Medical Sciences, Kumamoto, Japan
[4]Department of Neurosurgery, Hyogo College of Medicine, Hyogo, Japan

Running title
FXa induces smooth muscle cell senescence.

Address correspondence to:

Yoshiaki Taniyama, MD, PhD, Associate Professor

Department of Clinical Gene Therapy, Osaka University Graduate School of Medicine.

2-2 Yamada-oka, Suita, Osaka, 565-0871

Tel.: +81-6-6210-8351, FAX: +81-6-6210-8359

E-mail address; taniyama@cgt.med.osaka-u.ac.jp

Ryuichi Morishita, MD, PhD, Professor

E-mail: morishit@cgt.med.osaka-u.

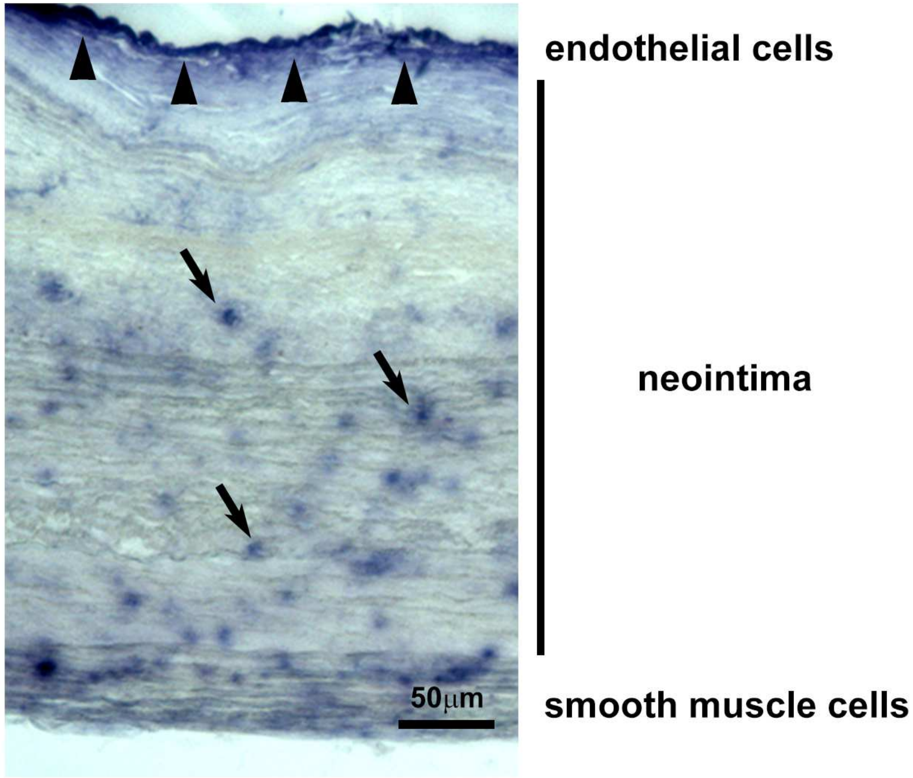

Supplementary figure 1
Factor X expression in human atherosclerotic plaques. Strong FX mRNA signal was detected in VSMCs, endothelial cells (arrow head) and inflammatory cells (black arrow).

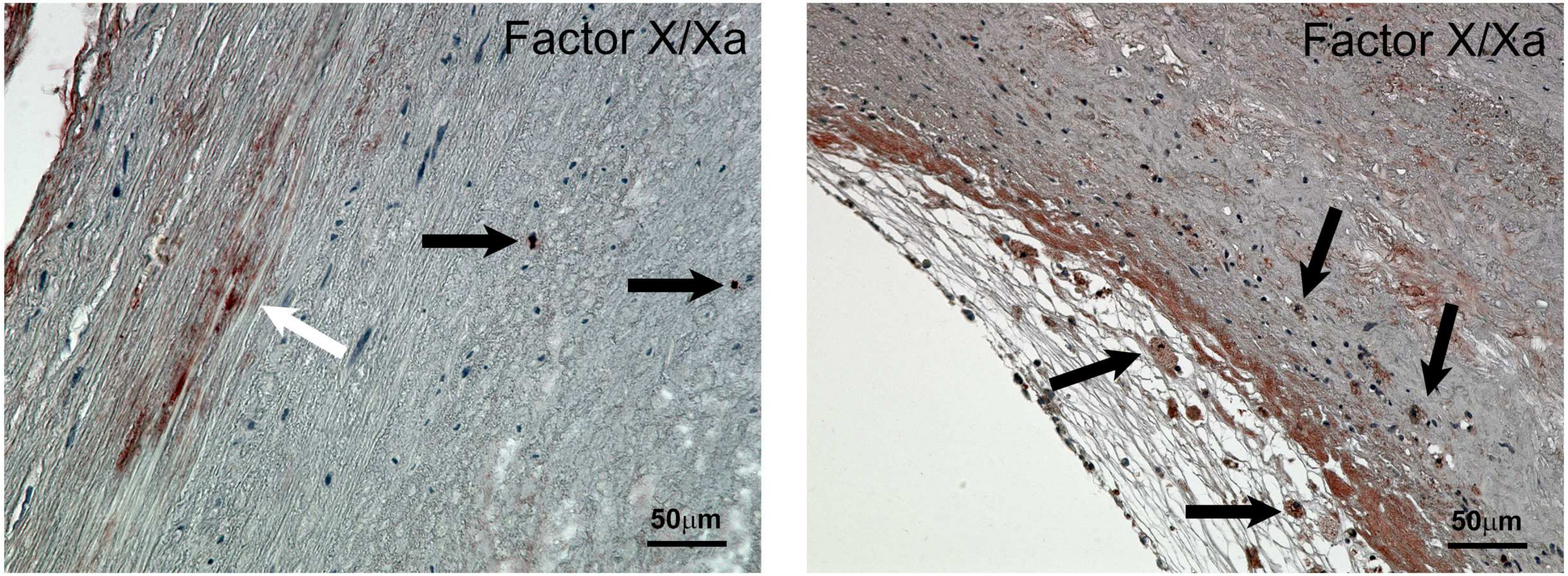

Supplementary figure 2

Factor X/Xa expression in human atherosclerotic plaques. Strong FX/FXa signals were detected in VSMCs (white arrow) and inflammatory cells (black arrow).

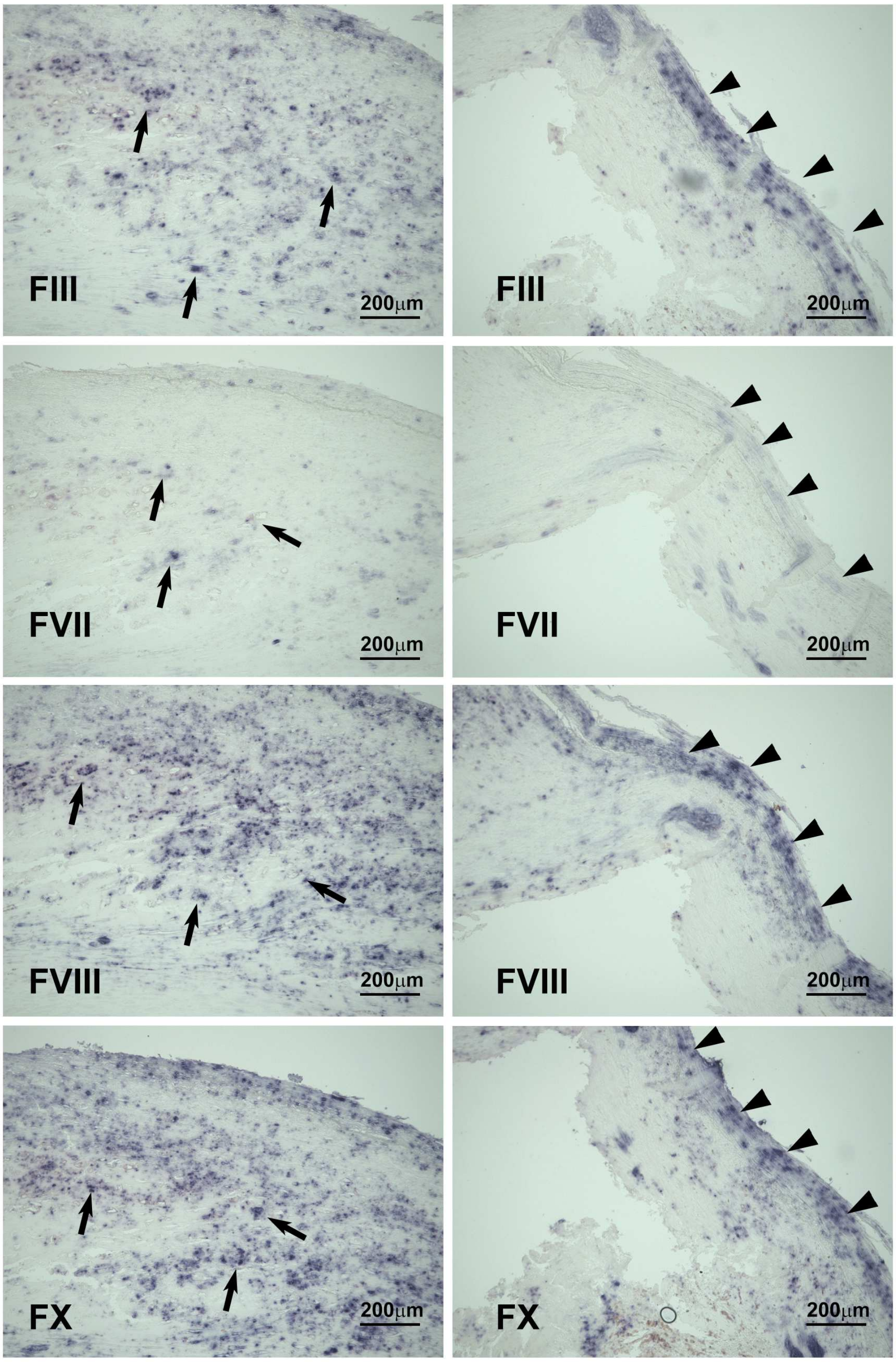

Supplementary figure 3

Factor III, VII, VIII, and X mRNA expression in human atherosclerotic plaques. Strong FIII, FVIII, and FX mRNA signals was detected in inflammatory cells (arrow head) andVSMCs(black arrow). FVII signal was relatively weak, but detected in VSMCs and inflammatory cells..

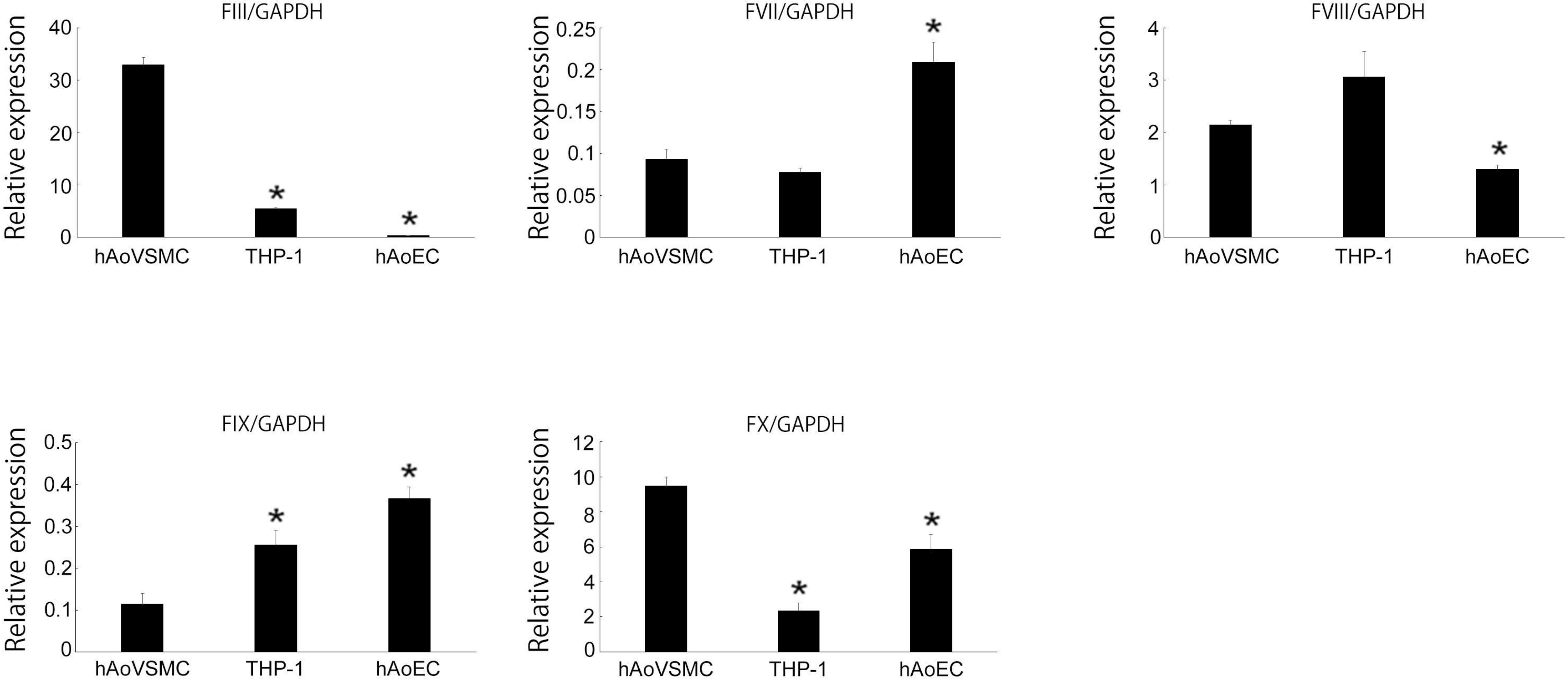

Supplementary figure 4 FIII, FVII, FVIII, FIX, and FX mRNA expression in hAoVSMC, THP-1, and hAoEC. *P<0.05 vs. hAoVSMC, n=5.

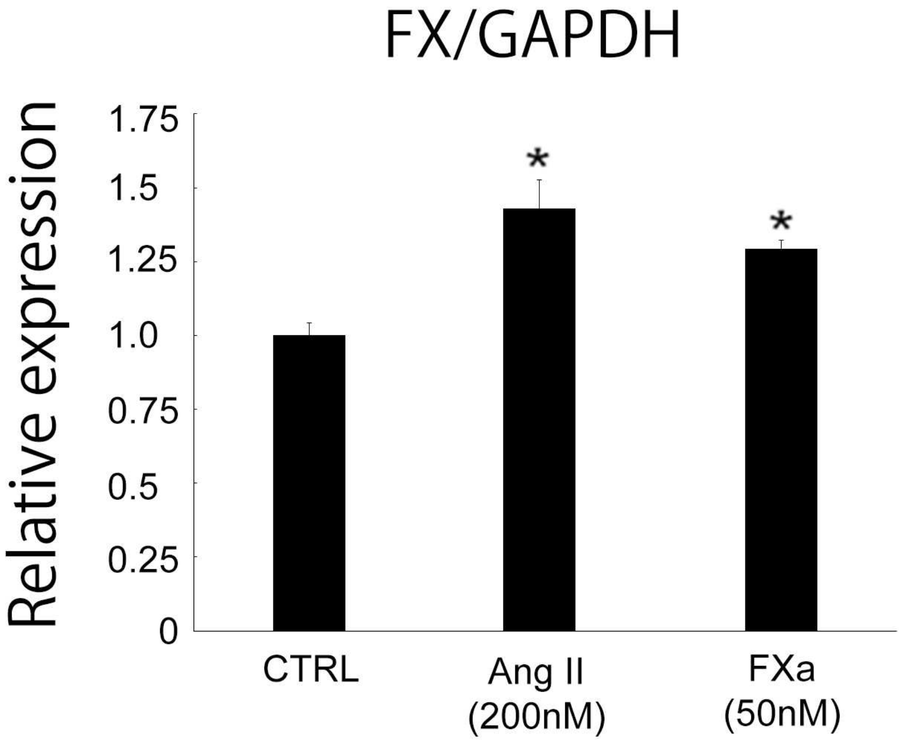

Supplementary figure 5 FX mRNA expression in human aortic VSMCs in vitro. Ang II = angiotensin II. *P<0.05 vs. control, n=6.
